# Human brucellosis in pregnancy in western Uganda: High seroprevalence and health risk factors identified in a cross-sectional study

**DOI:** 10.1371/journal.pntd.0014123

**Published:** 2026-03-20

**Authors:** Wainaina Virginia, Marie Pascaline Sabine Ishimwe, Ahmed Kiswezi Kazigo, Tshimanga Tshilumba, Jean de Dieu Rukamba, Theophilus Pius, Sawda Abdikarim Sheikh Isse, Theoneste Hakizimana

**Affiliations:** 1 Department of Obstetrics and Gynecology, Kampala International University, Ishaka, Uganda; 2 Department of Pediatrics and Child Health, Kampala International University, Ishaka, Uganda; 3 Department of Surgery, Kampala International University, Ishaka, Uganda; 4 Department of Laboratory sciences, Kampala International University, Ishaka, Uganda; University of Connecticut College of Agriculture Health and Natural Resources, UNITED STATES OF AMERICA

## Abstract

**Background:**

Human brucellosis is a neglected zoonosis that can cause anemia, miscarriage and pre-term birth, yet its burden during pregnancy in Uganda is unknown. This study determined the seroprevalence and associated risk factors for brucellosis among pregnant women at a Tertiary Hospital in Uganda.

**Methodology:**

This cross-sectional study was conducted in the antenatal clinic of Kampala International University Teaching Hospital, western Uganda (September–December 2024). Consecutive participants provided sociodemographic and livestock-exposure data and 5 mL of venous blood was drawn. Sera were screened with the Rose Bengal Plate Test; all reactive samples and 10% of non-reactive samples were confirmed with an indirect IgG/IgM ELISA. Multivariable logistic regression identified factors independently associated with seropositivity with significance set at P < 0.05.

**Results:**

Of 207 enrolled women (median gestation = 24 weeks), 29 were ELISA-confirmed seropositive, giving a prevalence of 14.0% (95% CI 9.2–18.8). Independent risk factors were lack of formal education (adjusted odds ratio [aOR] 4.05, 95% CI 1.02–16.01), consumption of fresh milk or under-cooked meat (aOR 5.70, 95% CI 1.94–16.76), frequent contact with animal manure (aOR 3.29, 95% CI 1.29–8.47) and rearing livestock at home (aOR 3.75, 95% CI 1.36–10.32).

**Conclusions:**

One in seven pregnant women in this mixed livestock–human ecosystem showed evidence of brucellosis, far above the WHO elimination threshold. Integrating RBPT-based screening into routine antenatal care, promoting milk pasteurization and safe meat preparation, improving manure handling and strengthening herd vaccination through One-Health collaboration could reduce maternal infection and adverse pregnancy outcomes in similar smallholder settings.

## Author summary

Brucellosis is a bacterial disease that spreads from livestock to people through unpasteurized milk, undercooked meat or direct animal contact. Although pregnancy is a vulnerable period, there is almost no information on how common the infection is among pregnant women in East Africa. We invited all women who attended the antenatal clinic of Kampala International University Teaching Hospital in rural western Uganda between September and December 2024 to take part in a one-time blood test and short interview. Using a two-step laboratory algorithm (Rose Bengal Plate Test followed by IgG/IgM ELISA), we found that 14% of participants had Brucella antibodies, indicating serological evidence of exposure. Women without formal schooling, those who drank fresh milk or ate under-cooked meat, those who handled animal manure and those who reared animals at home were three- to six-times more likely to be infected. The results highlight the need for antenatal screening, food-safety messages, and closer collaboration between veterinary and maternal-health services to protect mothers and babies in similar smallholder settings

## Introduction

Brucellosis is a neglected zoonotic infection that ranks among the seven priority bacterial diseases targeted by the World Health Organization’s neglected-tropical-diseases roadmap [1]. Persistent endemicity in sub-Saharan Africa reflects weak veterinary–public-health coordination, inadequate diagnostic capacity and limited community awareness, especially in low-income, smallholder settings [2]. Humans acquire infection mainly through consumption of unpasteurised dairy products, handling of infected animal tissues or inhalation of contaminated aerosols [3]. Pregnancy increases concern because clinical signs are non-specific and easily misattributed to common obstetric conditions, yet brucellosis has been linked to abortion, intra-uterine fetal demise, pre-term delivery, low birth weight and neonatal death [4].

Most prevalence data derive from mixed adult populations or high-risk occupational groups and show wide geographical variation. Community surveys have reported seropositivity ranging from 1.83% in rural India [5] and 3.7% among goat-exposed Thai women [6] to 23–31% in Egyptian pastoral communities [7] and 20% among livestock workers in Zambia [8]. Studies that focus specifically on pregnancy remain rare: a prospective Thai cohort found 3.7% seropositivity [6], Yemeni antenatal clinics reported 6–14% [9] and scattered African hospital studies show values from 0.28% in Cameroon [2] to 19% in Nigerian women with miscarriage [10]. The only pooled estimate for East Africa suggests a prevalence of 10.9% among pregnant women, but supporting primary data are sparse and heterogeneous [4]. Ugandan reports likewise vary from 4.4% seropositivity in Iganga District to 33% household exposure in Sheema [11] and no data are available for Bushenyi District, despite its dense human-livestock interface.

Risk factors identified across settings include low or no formal education, consumption of raw milk or under-cooked meat, direct contact with manure or abortive materials, and keeping livestock in household compounds [2,4,6,12,13]. Socio-economic constraints, poor hygiene practices and limited knowledge of zoonoses further amplify exposure [1]. Given the evident knowledge gaps and the potential for serious obstetric sequelae, district-specific data are essential to guide integrated One-Health interventions.

This study therefore assessed the seroprevalence of brucellosis and its associated behavioural, environmental and socio-demographic factors among pregnant women attending antenatal care in Bushenyi District, western Uganda. By providing the first pregnancy-focused brucellosis data for this setting, our findings aim to inform targeted screening strategies, risk-reduction messaging and cross-sectoral control programmes in similar smallholder communities across East Africa.

## Materials and methods

### Ethics statement

Ethical approval was obtained from Kampala international University (KIU) Research Ethics Committee (Reference number: KIU-REC-2024–398). Written informed consent was secured from all before enrolment. Seropositive women were counselled and referred for obstetric monitoring and appropriate antimicrobial therapy. All participants were aged 18 years or older and each provided consent personally. The research adhered strictly to ethical standards as outlined in the Declaration of Helsinki.

### Study design and setting

A cross-sectional survey was conducted between 1 September and 31 December 2024 in the antenatal clinic of Kampala International University Teaching Hospital (KIU-TH), Bushenyi District, western Uganda. The hospital is the district’s main referral centre, serving a mixed crop–livestock smallholder population in which raw-milk consumption and backyard cattle, goat and pig rearing are common. The hospital is comprised of different departments under different specialties offering both out-patient and in-patient services with a 700-bed capacity for the in-patients. The obstetrics and gynecology department where this study was conducted comprises one of the departments of the hospital. It operates both outpatient and inpatient services with a 75-bed capacity that caters to both obstetric and gynecological patients. Currently, there are five specialists, sixty resident doctors, one intern doctor, three intern nurses and six midwives. The antenatal unit receives appoximately10–15 patients per day from within the surrounding region. The majority of the patients are involved in animal and crop farming. The hospital has a fully functional modern referral laboratory that caters to cytological, histopathological, microbiological, hematological, and serological and biochemical testing.

### Participant recruitment and sample-size determination

All pregnant women attending routine antenatal care during the study period were consecutively enrolled to participate. All pregnant women attending routine antenatal care during the study period were eligible. Women were excluded if they were critically ill or unable to provide informed consent due to impaired mental status. Women who declined consent were not enrolled. No participant met the clinical exclusion criteria. The clinic’s 10–15 attendees/day represents total visits (including repeat follow-up visits). Recruitment was conducted on scheduled clinic days when study staff were present; eligible women were approached consecutively and only once. In total, 228 women were approached during the data-collection sessions, 207 consented and were enrolled.

Sample size for a single-proportion study was calculated using the standard formula for estimating a population proportion with specified absolute precision.


$$\text{n}=\frac{{\text{z}}^{\text{2}}\text{p }(\text{1}-\text{p})}{{\text{d}}^{\text{2}}}$$

We used an expected Brucella seroprevalence of 10.9% reported among pregnant women in Northern Tanzania (East Africa), with a 95% confidence level (Z = 1.96) and 5% precision (d = 0.05), giving 150 participants [4]. Allowing 10% for non-response and rounding up to improve stability for multivariable logistic regression, we targeted ≥200 enrolments and ultimately analysed 207 participants.

### Specimen collection and laboratory procedures

Five millilitres of venous blood were obtained aseptically into plain Vacutainer tubes, transported at ambient temperature and processed within 1 h at the KIU-TH serology laboratory. After clotting, samples were centrifuged at 4 000 rpm for 2 min and the serum was aliquoted into bar-coded cryovials; if immediate testing was not possible, aliquots were stored at 2–8 °C for less than 24 h.

Serum was first screened with the Rose Bengal Plate Test (RBPT; Institute Pourquier, France; lot RB25–0825). Reagents were equilibrated to 25–28 °C and 30 µL each of serum and antigen were mixed on a glass slide, rocked manually for 4 min, and read macroscopically; Agglutination was interpreted as reactive for anti-Brucella antibodies using the manufacturer’s antigen and reading criteria; as a serologic screening test, RBPT indicates Brucella spp. antibody reactivity and does not differentiate Brucella species. All RBPT-reactive sera and 10% of randomly selected RBT-non-reactive sera were confirmed with an indirect IgG/IgM ELISA (NovaLisa, NovaTec Immundiagnostica, Germany; lot 2025-04) following the package insert; results > 11 NovaTec units were positive, 9–11 equivocal (retested once), and < 9 negative. Published pooled accuracy estimates for these tests have been reported as: RBPT sensitivity 96.6% (95% CI 92.6–98.5) and specificity 97.9% (93.1–99.4); ELISA sensitivity 94.3% and specificity 98.6% [14].

### Quality control

Each RBPT and ELISA run incorporated the manufacturer’s positive/negative controls plus an in-house archived positive and negative serum. Every 20th specimen was split and blindly retested at Fort Portal Regional Referral Hospital, yielding 100% concordance. Eligibility criteria were applied strictly, data were collected with a single questionnaire pre-piloted on 20 antenatal clients, and forms were checked on-site and again at day’s end before entry.

### Statistical analysis

Data were entered in Microsoft excel version 16 and analysed with Stata 17 (StataCorp, College Station, TX, USA). Continuous variables were summarised by medians (IQR); categorical variables by frequencies and percentages. Bivariable logistic regression screened independent variables; those with p < 0.20 entered a multivariable model fitted with backward elimination. Multicollinearity was assessed with variance-inflation factors (VIF > 10 denoted collinearity); model fit was evaluated using the Hosmer–Lemeshow test. Adjusted odds ratios (aOR) with 95% confidence intervals and two-sided p-values < 0.05 were considered statistically significant.

## Results

### Characteristics of the study participants

**Fig 1:**
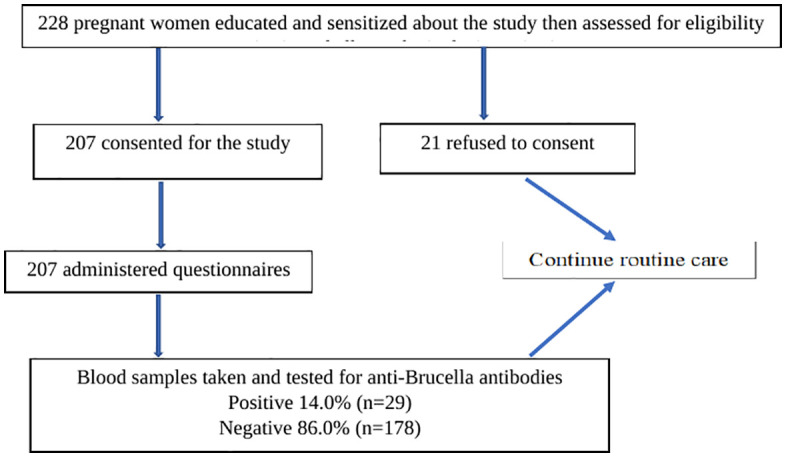
Study flow chart.

Of 228 eligible pregnant women approached, 207 consented to participate and provided blood samples for Brucella serologic testing (see Fig 1). The majority of the study participants were less than 30 years old 130(62.8%) and were from the Munyankole tribe 194(93.7%). A significant number of respondents had no formal education 83(40.1%), most were married 201(97.1%) and lived in urban areas 113(54.6%). Regarding occupation, most respondents were self -employed 141(68.1%) followed by formal employment 44(21.3%). The monthly income for the majority was less than 200,000 Ugandan Shillings 116(56.0%) (Table 1).

**Table 1:** Sociodemographic characteristics of the study participants (N = 207).

Variables	Categories	Frequency (n)	Percentage (%)
Age (Years)	≥30	77	37.2
	18-29	130	62.8
Religion	Christian	181	87.4
	Muslim	26	12.6
Tribe	Munyankole	194	93.7
	Other	13	6.3
Level of education	No formal education	83	40.1
	Primary	41	19.8
	Secondary	51	24.6
	Tertiary	32	15.5
Marital status	Single	6	2.9
	Married	201	97.1
Place of residence	Rural	94	45.4
	Urban	113	54.6
Occupation	Self- employed	141	68.1
	Unemployed	22	10.6
	Formally employed	44	21.3
Monthly Income (UGX)	< 200,000 UgX	116	56
	200,000 + UgX	91	44

UgX, Ugandan Shillings (Monthly Income).

### Seroprevalence of brucella infection among pregnant women seeking care at KIU-TH (N= 207)

**Fig 2:**
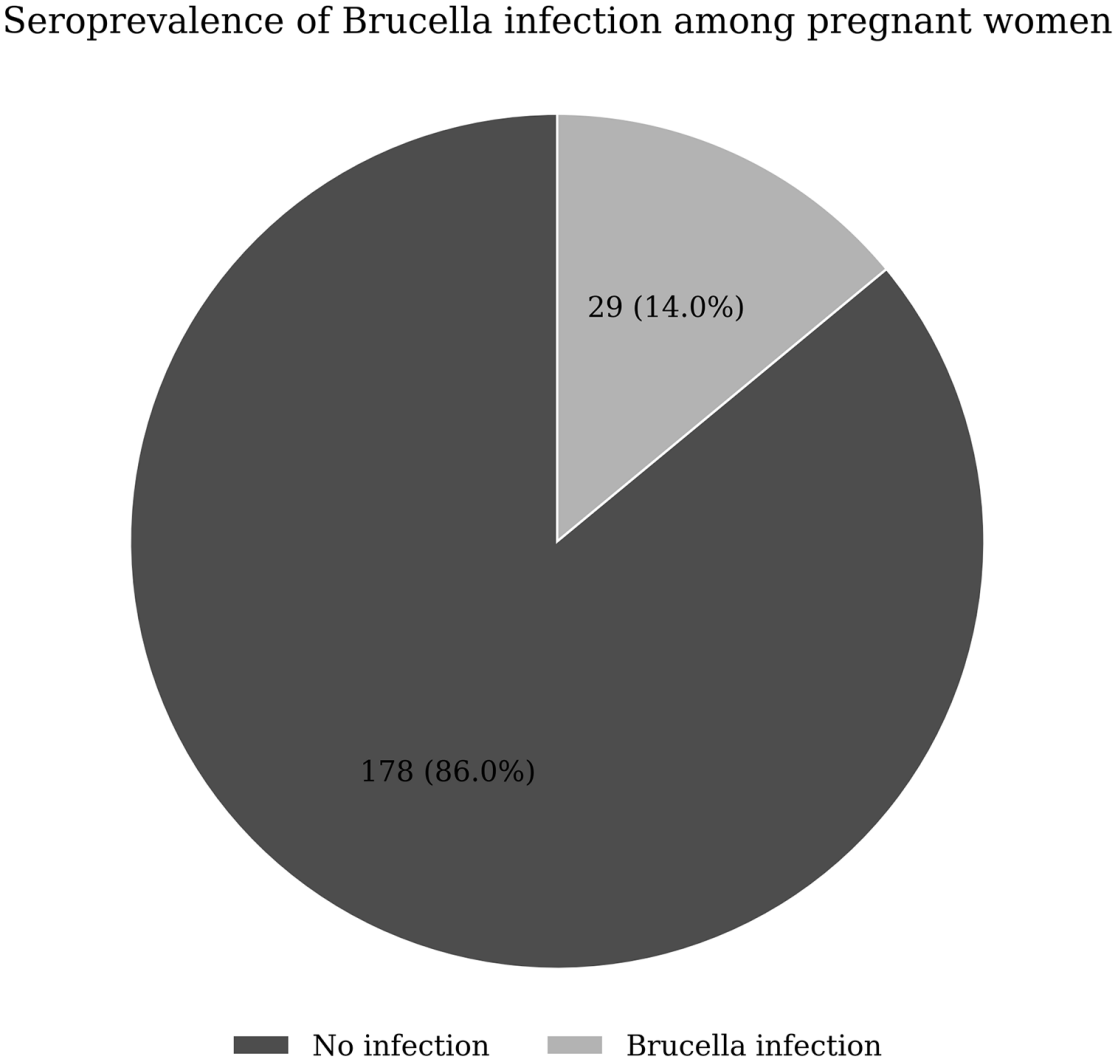
Seroprevalence of brucella infection among pregnant women seeking care at KIU-TH.

Of 207 enrolled women (median gestation = 24 weeks), 29 were ELISA-confirmed Brucella spp. seropositive, giving a prevalence of 14.0% (95% CI 9.24–18.77). The remaining 178/207 (86.0%) were ELISA-negative (seronegative) (Fig 2).

Using the two-step testing algorithm, 29 participants were RBT-reactive. Of these, 29 were ELISA-confirmed seropositive, giving an ELISA confirmation proportion of 29/29 (100%). In addition, ELISA testing of a random 10% subset of RBT-non-reactive samples (n = 18 out of 178 RBT-non-reactive) identified no ELISA-positive samples (0/18, 0%), suggesting minimal false-negative results among RBT-non-reactive samples within the subset tested.

### Factors associated with brucella infection among pregnant women seeking care at KIU-TH (N = 207)

In the bivariable analysis (Table 2), five factors such as level of education, marital status, ingestion of animal products, interaction with animal manure and rearing animals were associated with brucella infection among pregnant women seeking care at KIU-TH. At the multivariable level of analysis (Table 3), Brucella seropositivity remained independently associated with lack of formal education (aOR = 4.05; 95% CI 1.02–16.01; p = 0.046), ingestion of animal products (fresh milk or under-cooked meat (aOR = 5.70; 95% CI 1.94–16.76; p = 0.002), Interaction with animal manure (aOR = 3.29; 95% CI 1.29–8.47; p = 0.013) and rearing livestock at home (aOR = 3.75; 95% CI 1.36–10.32; p = 0.011).

**Table 2:** Bivariate analysis of sociodemographic, behavioural, obstetric and medical factors associated with brucella infection among pregnant women seeking care at KIU-TH.

Factors	Categories	*Brucella* results		cOR(95%CI)	P-value
**Negative**	**Positive**
**Age**	≥30	64	13	0.69(0.31-1.58)	0.361
	18-29	114	16	Ref	
**Religion**	Christian	154	27	0.48(0.11-2.13)	0.331
	Muslim	24	2	Ref	
**Tribe**	Munyankole	165	29		
	Other	13	0	Ref	
**Level of education**	No formal education	75	8	4.91(1.75-13.77)	0.002*
	Primary	35	6	3.06(0.99-9.48)	0.053*
	Secondary	47	4	6.16(1.76-21.58)	0.004*
	Tertiary	21	11	Ref	
**Marital status**	Single	4	2	0.31(0.05-1.78)	0.189*
	Married	174	27	Ref	
**Residence**	Rural	82	12	1.21(0.55-2.68)	0.639
	Urban	96	17	Ref	
**Occupation**	Self-employed	121	20	1.15(0.45-2.92)	0.777
	Unemployed	20	2	1.89(0.36-9.98)	0.452
	Formally employed	37	7	Ref	
**Monthly income (UgX)**	< 200,000 UgX	101	15	1.22(0.56-2.69)	0.614
	200000+ UgX	77	14	Ref	
**Ingestion of animal products**	Yes	84	24	5.37(1.96-14.71)	0.001*
	No	94	5	Ref	
**Interaction with animal manure**	Yes	54	15	2.46(1.11-5.45)	0.027*
	No	124	14	Ref	
**Rearing animals**	Yes	80	21	3.22(1.35-7.65)	0.008*
	No	98	8	Ref	
**Gravidity**	Multiparous	145	24	0.92(0.33-2.58)	0.867
	Primiparous	33	5	Ref	
**History of adverse obstetric outcomes**	Yes	45	6	0.87(0.37-2.15)	0.792
	No	133	23	Ref	
**HIV status**	Positive	17	5	0.51(0.17-1.50)	0.220
	Negative	161	24	Ref	

*p≤0.2, cOR = Crude odds ratio, CI =Confidence interval.

**Table 3:** Multivariate analysis of factors associated with brucella infection among pregnant women seeking care at KIU-TH.

Factors	*Brucella* results		cOR (95%CI)	P-value	aOR (95%CI)	P -value
**Negative**	**Positive**
**Level of education**						
No formal Education	75	8	4.91(1.75-13.77)	0.002	4.05(1.02-16.01)	0.046**
Primary	35	6	3.06(0.99-9.48)	0.053	1.47(0.33-6.67)	0.615
Secondary	47	4	6.16(1.76-21.58)	0.004	3.73(0.77-18.04)	0.102
Tertiary	21	11	Ref		Ref	
**Marital status**						
Singled	4	2	0.31(0.05-1.78)	0.189	0.25(0.01-4.57)	0.351
Married	174	27	Ref		Ref	
**Ingestion of animal products**						
Yes	84	24	5.37(1.96-14.71)	0.001	5.70(1.94-16.76)	0.002**
No	94	5	Ref		Ref	
**Interaction with animal manure**						
Yes	54	15	2.46(1.11-5.45)	0.027	3.29(1.29-8.47)	0.013**
No	124	14	Ref		Ref	
**Rearing animals**						
Yes	80	21	3.22(1.35-7.65)	0.008	3.75(1.36-10.32)	0.011**
No	98	8	Ref		Ref	

aOR = adjusted odds ratio; CI = confidence interval; UGX = Uganda shillings; Ref = reference category. **p < 0.05.

## Discussion

The present study showed a Brucella seroprevalence of 14% among antenatal attendees in Bushenyi District, a level that places pregnant women in this mixed crop–livestock ecosystem well above the 1% elimination threshold proposed by the WHO. A similar prevalence was reported from Yemeni antenatal clinics (13.82%) [9] and in a Tanzanian cohort drawn from small-ruminant-keeping areas (16.2%) [15], suggesting that where subsistence animal husbandry, raw‐milk consumption and limited veterinary services coexist, brucellosis poses a consistent maternal risk. The modest difference from the Tanzanian estimate may reflect our smaller sample and the use of a two-step algorithm that mitigates false-positive Rose Bengal results.

Considerably higher seroprevalence has been documented among Kenyan pastoralists (54%) [16], Nigerian women with miscarriage (19%) [10] and Zambian abattoir workers (20%) [8]. These studies purposely enrolled highly exposed or symptomatic sub-groups and, in some cases, relied on single agglutination tests prone to cross-reactions, factors that inflate prevalence compared with a general antenatal population screened by RBT plus ELISA. Conversely, urban or molecular surveys, such as the qPCR study in Pakistan (4.4%) [17] and the rural Indian serosurvey (1.83%) [5], yielded lower estimates, underscoring how geography, diagnostics and lifestyles shape the apparent burden.

Four modifiable exposures remained independently associated with infection. Women without formal schooling had four-fold higher odds of seropositivity, mirroring reports from Cameroon and India that link low education to limited knowledge of zoonoses and fewer hygienic barriers [2,5]. Regular consumption of unpasteurised milk or under-cooked meat increased risk almost six-fold, a pathway well documented in East African pastoral settings [4,6]. Frequent contact with animal manure tripled the odds of infection, likely because *Brucella* can persist in dried excreta and contaminate household soil and dust. This finding was in agreement with the findings of Ngorongoro, Makala et al., who reported Brucella infection among pregnant women who were in close contact with livestock [4]. Finally, keeping livestock at home raised the adjusted odds nearly four-fold, reinforcing the One-Health observation that household herd status predicts human seropositivity in Uganda and Kenya [11,16]. Additionally, Kledmanee et al., reported that the seroprevalence of brucellosis among women rearing goats or having neighbors rearing goats were more likely to be exposed to Brucella [6]. We interpret socio-demographic variables such as education as potential proxy indicators of knowledge, food-handling practices, and intensity of livestock exposure rather than direct causal factors. The more proximal and biologically plausible pathways in our setting remain consumption of unpasteurized dairy/undercooked meat and frequent household contact with livestock or their waste

## Study strengths and limitations

This study provides early evidence on Brucella spp. seropositivity and associated factors among pregnant women attending antenatal care in Western Uganda, an under-studied population. Use of a standardized questionnaire and a two-step testing algorithm (RBPT screening with ELISA confirmation) strengthened internal consistency and supported awareness of zoonotic risk during ANC services. Interpretation should consider key limitations. The cross-sectional design precludes causal inference and does not establish temporality. The tests detect anti-Brucella antibodies, indicating exposure rather than confirmed active infection, and do not differentiate Brucella species (culture/PCR was not performed). Although all RBT-reactive samples were ELISA-confirmed and none of the tested RBPT-non-reactive samples (random 10% subset) were ELISA-positive, some misclassification remains possible. Pregnancy outcomes were not systematically collected, limiting assessment of maternal or neonatal outcomes. Finally Sample size was calculated using an expected prevalence from an ANC-based pregnant population as our target participants; however, higher seroprevalence has been reported in nearby cattle-keeping household populations in Sheema District. If the true ANC prevalence were closer to these higher estimates, our sample size may be underpowered for some associations and the prevalence estimate may be less precise

## Conclusions and recommendations

Approximately one in seven pregnant women attending antenatal care in Bushenyi had ELISA-confirmed Brucella spp. seropositivity, indicating substantial exposure in this setting. Seropositivity was associated with low education, consumption of unpasteurized milk and/or undercooked animal products, frequent contact with animal manure, and household livestock rearing. Given the cross-sectional design and serologic methods (which indicate exposure rather than species-confirmed active infection), we recommend strengthening ANC-based risk assessment and targeted health education on safe food handling and safer animal-waste practices, alongside One Health measures such as improved zoonotic surveillance and livestock vaccination programs to reduce exposure in similar smallholder communities.

## Supporting information

S1 Data De-identified dataset used for analyses. (XLSX)
